# Association of Low Expression of *NUMB* in Peripheral Blood with Acute Myocardial Infarction

**DOI:** 10.1155/2022/7981637

**Published:** 2022-04-26

**Authors:** Heyu Meng, Lihong Li, Jianjun Ruan, Yanqiu Chen, Zhaohan Yan, Jinsha Liu, Xiangdong Li, Cuiying Mao, Ping Yang

**Affiliations:** ^1^Jilin Provincial Precision Medicine Key Laboratory for Cardiovascular Genetic Diagnosis, Changchun 130033, China; ^2^Jilin Provincial Engineering Laboratory for Endothelial Function and Genetic Diagnosis of Cardiovascular Disease, Changchun 130033, China; ^3^Jilin Provincial Molecular Biology Research Center for Precision Medicine of Major Cardiovascular Disease, Jilin Provincial Cardiovascular Research Institute, Changchun 130033, China; ^4^Department of Cardiology, China-Japan Union Hospital of Jilin University, Changchun 130033, China

## Abstract

**Objective:**

Our study's goal was to find out acute myocardial infarction (AMI) patients' *NUMB* gene expression patterns and to evaluate its role as a diagnostic marker for AMI detection.

**Methods:**

Peripheral blood was drawn from 124 individuals who had an AMI and 115 patients who had stable coronary artery disease (SCAD). The real-time quantitative polymerase chain reaction was used to measure the mRNA expression level of the *NUMB* gene in peripheral blood.

**Results:**

The AMI group's *NUMB* gene expression was 0.906 (0.181–0.954), whereas the SCAD group's expression was 1.024 (0.207–1.127). However, the AMI group had 0.885 times lower *NUMB* mRNA expression than the SCAD group (*P* < 0.05).

**Conclusion:**

Multivariate logistic regression evaluation found that lower *NUMB* expression was correlated with an increased risk of coronary artery disease. However, age and fasting plasma glucose levels were not associated with decreased *NUMB* expression.

## 1. Introduction

Although cardiovascular disease mortality is declining, it is still the leading cause of death globally [1]. About 20% of all fatalities in Europe and the United States are attributed to coronary artery disease (CAD), which kills more than 1.7 million people annually. IHD is still the largest cause of death in the world, despite a 22% drop in mortality from ischemic heart disease (IHD) during the previous 30 years [2]. One of the major causes of mortality in IHD is acute myocardial infarction (AMI). The incidences of AMI and post-AMI mortality are declining in most countries, especially in developed countries with high per capita income [3–5].

Treatment options such as coronary intervention, coronary bypass surgery, and drugs have improved the prognosis of AMI over the past few decades. However, the reduction in mortality has, at best, remained modest. Therefore, early identification and risk stratification of AMI is important to accelerate early intervention [6, 7]. Although highly sensitive biomarkers such as troponin T and I and creatine kinase-MB have long been used in a clinical setting to detect and diagnose AMI, the emergence of novel biomarkers may considerably improve the accuracy of early diagnosis. Advances in genome-wide analysis, especially microarray analysis, play an important role in discovering the novel clinical biomarkers of AMI [8, 9]. Whole blood gene expression profiling can provide information on the dynamics of disease states and shed light on the underlying disease mechanisms [10]. Clinically, many common diseases such as AMI [11–14] and different types of atherosclerosis [15] exhibit a characteristic gene expression profile.

First discovered in Drosophila, *NUMB* is an endocytic adaptor protein [16] that has been extensively studied as an inhibitor of the Notch receptor signaling pathway [17]. *NUMB* is an evolutionarily conserved protein that regulates endocytosis, adhesion, cell division, cell fate, migration, various signaling pathways (i.e., Notch, Hedgehog, and p53), and ubiquitination of certain substrates [18]. According to recent research, inhibition of the aforementioned *NUMB*-dependent processes is important in cancer treatment in addition to controlling physiological developmental processes. For instance, *NUMB* is significantly expressed in cervical squamous cell carcinoma [19] and astrocytoma [20], while downregulated in salivary gland cancer [21], non-small-cell lung cancer [22], and breast cancer [23]. However, no study has been performed on the correlation between AMI and *NUMB* gene expression. According to recent research, it has been involved in the JIP2/LAMP1/JNK pathway's control of ischemia-reperfusion. The expression of *NUMB* can improve myocardial injury and left ventricular function and reduce cardiomyocyte apoptosis [24].

Gene chip detection was performed in our prior work, and we observed that the AMI group's peripheral blood *NUMB* gene expression levels were significantly lower compared to the control group. This study, therefore, aims to validate the findings of the gene chip detection of *NUMB* expression and to investigate whether the *NUMB* gene may be employed as a molecular marker for the early identification of AMI.

## 2. Materials and Methods

### 2.1. Study Technology Route

**Figure 1:**
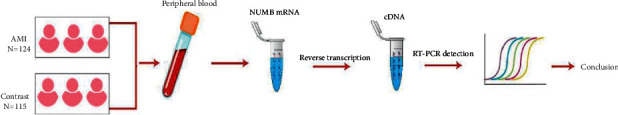
Schematic diagram depicting the research technology route. See research subjects and methods for details.

The overall methodology used in this study is depicted in Figure 1. There were 239 patients, 124 among them participated in the experimental group and 115 of whom participated in the control group. RT-qPCR was used to evaluate the relative mRNA expression level of total RNA collected from peripheral blood. SPSS software was used to conduct the statistical analysis.

### 2.2. Research Subjects

All study participants were admitted to the Department of Cardiovascular Medicine at the China-Japan Union Hospital of Jilin University from March to May 2018. The experimental group consisted of 124 individuals who had been recognized as having AMI in accordance with the 2018 update to the global definition of MI [25]. If there was clinical evidence of acute myocardial damage and acute myocardial ischemia, the inclusion criterion for the AMI experimental group would be based on an increase and/or decrease in cTn values, at least one of which was greater than the upper reference limit of 99%. The presence of at least one of the following signs or symptoms is considered clinical evidence of acute myocardial ischemia, such as pathological Q wave development on new electrograms, coronary angiography or autopsy confirming the existence of coronary-arterial thrombosis (CAT/PAT), new ischemic electrocardiogram alterations, and imaging data revealing a new loss of myocardial activity or new regional wall motion abnormalities, respectively. Moreover, the control group consisted of 115 SCAD patients as per the 2019 ESC Guidelines for the Diagnosis and Management of Chronic Coronary Syndrome [26], based on the following inclusion criteria, i.e., patients with new heart failure or heart disease, patients with “stable” angina and dyspnea, patients who have been asymptomatic or had stable symptoms for more than a year after their initial diagnosis of coronary heart disease or after their most recent revascularization, patients who were recently revascularized or were asymptomatic and asymptomatic people with coronary heart disease who were found while screening for angina pectoris, probable vasospasm, or microvascular disease. Detailed clinical data, including gender and age, low-density lipoprotein, hypertension and diabetes, triglyceride, troponin levels, high-density lipoprotein, total cholesterol, and smoking history of all individuals, were recorded. Patients' informed permission was obtained before any samples or clinical data could be gathered from them.

The following were the exclusionary criteria: coronary artery bypass surgery (CABG) or percutaneous coronary intervention (PCI) may cause myocardial infarction. Second, MI is associated with a blood supply imbalance. Third, MI with cardiac or noncardiac surgery; fourth, multiple factors or unknown myocardial damage caused by uncertain diseases such as severe stress cardiomyopathy, severe pulmonary embolism, heart failure, or pulmonary hypertension, serious infectious diseases, malignant tumor complications, and other severe neurological diseases.

### 2.3. Study Procedures

#### 2.3.1. Harvesting of Lymphocytes

Six millilitres of blood from each individual were taken in the morning and kept at 4°C in EDTA anticoagulation tubes. A peripheral blood lymphocyte isolation kit was used to separate lymphocytes within four hours after sample collection. The equal volume of anticoagulant mixed with 0.9% NaCl was added to an equal volume of lymphocyte-isolated medium. The plasma layer, clear separation red blood cell layer, and milky white lymphocyte layer were separated from the sample after centrifugation at 3000 rpm for 20 minutes. After aspirating the lymphocyte layer, it was utilized in subsequent studies.

#### 2.3.2. Lymphocyte cDNA Synthesis

To extract total RNA, we used an extraction kit for blood total RNA (Blood Total RNA Kit, Xinjing Biological Reagent Development Co., Ltd., Hangzhou). To prevent RNA degradation or contamination, the extraction procedure was carried out in accordance with the kit's instructions. Polyacrylamide gel electrophoresis was used to check the quality of the RNA solution, and the visible brightness of the 28S rRNA band was nearly twice that of the 18S rRNA band. A microplate reader was used to measure the concentration and absorbance of RNA from samples that met the standards. The concentration and absorbance of RNA samples meeting the standards were determined using a microplate reader. The 260/280 value should be 1.7–2.1, and the A260/A230 value is >2. RNA samples were reverse transcribed following the manufacturer's instructions and satisfying the specifications of the reverse transcription kit (rapid one-step genomic cDNA first-strand synthesis premix, Tiangen Biochemical Technology Co., Ltd., Beijing). Each sample had the same RNA concentration. For the following phase of the investigation, a fluorescent quantitative polymerase chain reaction (PCR) was performed on the cDNA samples stored at –80°C.

#### 2.3.3. Real-Time Quantitative PCR (RT-qPCR)

An SYBR RT-qPCR kit (Sangon Fluorescence Quantitation Kit, Taq qPCR Synthesis Premix Reagent, Shanghai) was used to amplify the cDNA samples after a 20-fold dilution. As previously mentioned, the 20 *μ*L reaction solution included the following components: 10 *μ*L of 2 × SG Fast qPCR reaction mix; 2 *μ*L of DNF buffer; 0.4 *μ*L each of the forward and backward primers (concentration 10 *μ* mol/L); 6.2 *μ*L of sterilized double-distilled water; and 1 *μ*L of the cDNA sample. Furthermore, predenaturation at 95°C for 5 minutes was followed by 40 cycles of denaturation at 95°C for 3 s, annealing at 60°C for 30 s, and extension at 72°C for 20 s under the reaction conditions described previously. The melting and amplification curves in the 60°C to 95°C temperature range were recorded after the process. Based on the ABI-FAST7500 dissociation curve, amplification conditions were found to be highly specific for GAPDH and *NUMB*, respectively. All samples are represented as the relative expression level 2^−ct^2^−Δct^ (Δct = target gene ct value−reference gene ct value), which is the difference between the target gene's and the reference's cycle thresholds (ct).  Table 1 lists the primers used in this investigation.

### 2.4. Statistical Analysis

SPSS 25.0 was used to conduct the statistical analysis. The measurement data were subjected to a normality test. *X* ± *S* was used to statistically characterize data that followed a normal distribution (*P* > 0.1), and the differences between groups were examined using a two independent samples *t*-test. The median and interquartile ranges were used to statistically characterize data that did not follow a normal distribution (*P* ≤ 0.1), and differences between two independent groups were evaluated using the nonparametric rank-sum test. For statistical analysis, count data were characterized by differences between groups, and frequency was examined using the chi-square(*x*^2^) test. AMI-related risk variables were studied using binary logistic regression analysis. The link between *NUMB* gene expression and troponin I was studied using bivariate correlation analysis. At a two-sided test *P* ≤ 0.05, statistical findings were considered statistically significant.

## 3. Results

### 3.1. Clinical Data Analysis of Research Subjects

AMI patients were found to be considerably older than those in the control group (*t* = −2.318, *P*=0.020) and have significantly higher fasting plasma glucose levels (*Z* = −2.505, *P*=0.012) after clinical data analysis. However, there were no significant variations in the other indices (Table 2).

### 3.2. RT-qPCR for the Detection of *NUMB* Amplification Products

It was found that the amplification curve of the *NUMB* gene exhibited an apparent and smooth “S shape” utilizing peripheral blood RNA. There was only a single peak in the dissociation curve, and the amplification product showed a high level of specificity.

### 3.3. The mRNA Levels of the *NUMB* Gene Comparison between the AMI and SCAD Groups

**Figure 2:**
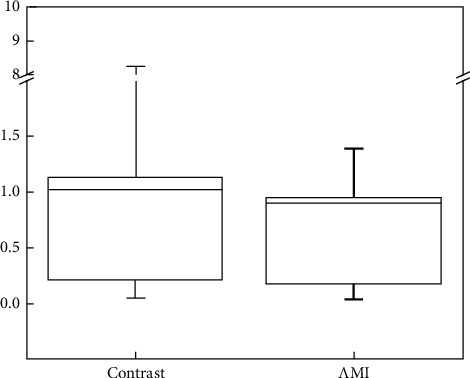
Relative expression of *NUMB* gene mRNA. The abscissa is a grouping, the ordinate is a relative expression, and the ordinate is an unequal distance coordinate. AMI, acute myocardial infarction.

The Δct values were obtained using RT-qPCR represent the average of 3 replicate measurements per sample. The results showed that the 2^−Δct^ of the AMI group was 0.906 (0.181–0.954) and that of the stable CAD group was 1.024 (0.207–1.127). There was a significant difference between the two groups (*P* < 0.05). The mRNA level of *NUMB* gene expression in the peripheral blood of patients in the AMI group was significantly lower than that of patients in the SCAD group, with a relative expression that was 0.885 times that in the SCAD group (Figure 2).

### 3.4. The Correlation Analysis between *NUMB* Gene Expression Levels and Patient Features

Our results demonstrated variations in the expression of the *NUMB* gene, age, and fasting plasma glucose levels between the AMI and SCAD groups. We also investigated to observe if mRNA expression of the *NUMB* gene was associated with fasting plasma glucose levels or age [27]. Subjects were divided into two groups based on their fasting plasma glucose levels, with the normal group having a level (≤5.6 mmol/L) and the increased group having a level >5.6 mmol/L. The standard age grouping split the participants into older (65 years of age and older) and younger (65 years of age and younger) groups. For each individual, the mRNA expression of the *NUMB* gene was expressed as 2^−Δct^ ratio, and the association between each group and *NUMB* expression was examined accordingly. The results showed no differences in the expression of the *NUMB* gene mRNA between the fasting plasma glucose normal and elevated groups (*P*=0.551). Additionally, no change was observed in the *NUMB* gene mRNA expression between the young and elderly (*P*=0.645) (Table 3).

### 3.5. Logistic Regression Analysis

The cutoff values for the relative expression of the *NUMB* gene were used to separate all participants into high (2^−Δct^ > 0.662) or low gene expression groups (2^−Δct^ ≤ 0.662). Based on the results of the binary logistic regression analysis, we were able to determine how age, fasting plasma glucose, and AMI all correlate with the mRNA expression of the *NUMB* gene as shown in Table 4(*P*=0.007). Decreased *NUMB* gene expression was an independent risk factor for AMI. Low *NUMB* gene expression increased the risk of AMI by 3.287 times when compared to high *NUMB* gene expression. In addition, older age (*P*=0.030, Table 4) was an independent risk factor for AMI by 1.853 times. Moreover, elevated fasting plasma glucose levels were not an independent risk factor for AMI (*P*=0.098, Table 4).

### 3.6. Bivariate Correlation

The AMI group had a troponin I concentration of 1.390 ng/mL (0.060–2.390). Myocardial infarction is measured by troponin I concentration. *NUMB* gene expression in peripheral blood does not have any correlation with serum troponin I concentration in a bivariate correlation analysis (*r* = −0.027, *P*=0.707), indicating there was no correlation between the expression level of *NUMB* gene in peripheral blood and the severity of acute myocardial infarction.

## 4. Discussion

In this study, we compared the expression of *NUMB* gene mRNA in peripheral blood between the AMI and SCAD groups and found that *NUMB* gene expression in the AMI group was significantly lower than that in the SCAD group and that its relative expression was 0.885 times that in the SCAD group.

Atherosclerosis is a pathological condition in which monocytes and lymphocytes adhere, migrate, and aggregate under the damaged intima to form foam cells due to intimal injury, which further leads to the development of lipid streaks in atherosclerotic plaque lesions [28]. Atherosclerosis is the most prevalent cause of CAD, which is also a widespread disorder that poses a major danger to human health globally. Studies have shown that the endocytic adaptor protein *NUMB* plays an important role in migrating cell-directed integrin transport mechanism [29]. Integrins are the main family of migration-promoting receptors that regulate cell migration and promote the development of atherosclerosis by mediating cells and the extracellular matrix [30], whereas integrin-stimulated cell migration requires the participation of *NUMB* [29]. However, to our knowledge, the role of the *NUMB* gene has not yet been studied in correlation to cardiovascular disease development and progression.

Studies have shown a strong, positive, independent relationship between serum total cholesterol and the incidence of CAD across a wide range of cholesterol levels, including normal and mildly elevated levels [31]. Lowering plasma cholesterol levels by inhibiting the absorption of exogenous cholesterol may prevent the development of atherosclerotic cardiovascular disease [32]. *NUMB* is a key regulator of cholesterol homeostasis in humans, mediating cholesterol absorption in the stomach and hepatic bile reabsorption in the liver [33]. Ablation of *NUMB* in the mouse intestine significantly reduces the absorption of dietary cholesterol and decreases plasma cholesterol levels [34]. Although overall blood cholesterol levels did not vary significantly between the two groups, we discovered that the level was greater in the low expression *NUMB* group. As a consequence of acute inflammation's inhibitory influence on lipoprotein concentrations [35], serum cholesterol levels decreased by 10.6% from days 1 to 2–4 after AMI by a mean of 0.55 mmol/L [36].

Fasting plasma glucose levels in the AMI and SCAD groups were substantially different, i.e., the AMI group was significantly higher than that of the SCAD group. Furthermore, there was no correlation observed between low *NUMB* gene expression and high levels of fasting plasma glucose. Although logistic regression analysis found that an increase in fasting plasma glucose levels was not an independent risk factor for AMI, the OR was still 1.619 (*P* > 0.05). We hypothesized that the stress reaction after an AMI may have triggered an increase in fasting plasma glucose levels.

AMI mortality and prevalence have been shown to rise with age [37]. In patients with AMI, age is a significant independent predictor of death in the hospital [38]. This research found that the AMI and SCAD groups were significantly different in age. Furthermore, we discovered that aging did not affect the *NUMB* gene's level of expression. Low *NUMB* gene expression was shown to be an independent risk factor for AMI in a binary logistic analysis (OR: 3.287, *P*=0.007), as was age (OR: 1.853, *P*=0.030). Patients with stable CAD having a low expression of the *NUMB* gene had 3.287 times the risk of an AMI, regardless of age or other variables. Elderly people have a 1.853-fold greater chance of having a heart attack. Therefore, it is speculated that elderly patients with lower *NUMB* expression are more prone to AMI.

Myocardial filaments and skeletal muscle contain troponin complexes. The troponin subunits I, T, and C make up this protein. Involved in muscle function, it relates changes in intracellular Ca^2+^ concentration to contraction. Troponin I and T have been frequently employed in the diagnosis of cardiomyocyte mortality (myocardial trauma, myocardial infarction, etc.) over the last 35 years [39]. The relationship between *NUMB* and troponin I was established by CTRP3. The downregulation of CTRP3 will lead to the upregulation of troponin I and increase the injury of myocardial ischemia-reperfusion. Research shows that *NUMB* is important in the control of ischemia-reperfusion in the LAMP1/JIP2/JNK pathway [24].

Our study has some shortcomings. Chemotaxis, adhesion, aggregation, lipid metabolism, and other biological processes of cells in the human body are regulated by various factors. The combined analysis of multiple genes and the overall study of each influencing factor may provide sufficient evidence for the genetic diagnosis and treatment of AMI. Although this study does not prove whether the low expression of *NUMB* is directly related to the occurrence of AMI, it can be speculated that the low expression of *NUMB* is one of the reasons for the occurrence of AMI. Therefore, we believe that prospective studies are an effective means of verifying a direct relationship.

## 5. Conclusions

Precisely, individuals with AMI had lower levels of *NUMB* gene expression in their peripheral blood than patients with SCAD. Peripheral blood *NUMB* gene expression was associated with an increased risk of acute myocardial infarction (AMI). *NUMB* gene expression in peripheral blood may be a molecular diagnostic for early detection of AMI.

## Figures and Tables

**Table 1:** RT-qPCR primer sequences.

Genes		Genes primer sequence (5′-3′)
*NUMB*	F^a^	TCAGCAGATGGACTCAGAGTT
	R^b^	AGGCTCTATCAAAGTTCCTGTCT

*GAPDH*	F^a^	TGTGGGCATCAATGGATTTGG
	R^b^	ACACCATGTATTCCGGGTCAAT

F^a^: forward primer; R^b^: reverse primer.

**Table 2:** Clinical data analysis of subjects in the AMI and SCAD groups.

Clinical indicators	AMI group (*N* = 124)	SCAD group (*N* = 115)	t/*x*^2^/*z*	*P*
Sex				
Male, *n* (%)	85 (68.6)	84 (73.0)	0.582	0.445
Female, *n* (%)	39 (31.4)	31 (27.0)		
Age	65.000 (57.000–74.000)	61.660 ± 8.778	−2.318	0.020
Hypertension, *n* (%)	57 (0.460)	58 (0.504)	0.477	0.490
Smoking history, *n* (%)	56 (0.452)	48 (0.417)	0.284	0.594
Type 2 diabetes, *n* (%)	38 (0.306)	28 (0.243)	1.184	0.277
TG (mmol/L)	1.570 (1.115–2.503)	1.660 (1.200–2.450)	−0.433	0.665
TC (mmol/L)	4.515 (3.810–5.188)	4.303 ± 0.992	−1.784	0.074
HDL-C (mmol/L)	0.950 (0.833–1.138)	0.971 ± 0.219	−0.706	0.480
LDL-C (mmol/L)	3.041 ± 1.011	2.817 ± 0.829	1.793	0.074
Fasting plasma glucose (mmol/L)	6.430 (5.510–9.280)	5.725 (5.198–7.375)	−2.505	0.012

**Table 3:** Correlation analysis of *NUMB* gene expression with fasting plasma glucose level and age.

Groups	No	*NUMB* relative expression	*Z*	*P*
Fasting plasma glucose normal	81	0.939 (0.775–1.281)	−0.596	0.551
Fasting plasma glucose elevated	138	1.015 (0.775–1.480)		
Younger age	132	1.001 (0.734–1.407)	−0.461	0.645
Older age	107	1.007 (0.796–1.500)		

**Table 4:** Logistic regression analysis of the independent risk factors for AMI.

	B	SE	WALD	Degrees of freedom	*P*	OR	95%CI
Low expression of *NUMB* gene	1.190	0.437	7.404	1	0.007	3.287	1.395–7.744
Fasting plasma glucose elevated	0.482	0.291	2.744	1	0.098	1.619	0.915–2.865
Older age	0.617	0.283	4.737	1	0.030	1.853	1.063–3.230

## Data Availability

The PCR data used to support the findings of this study are available from the corresponding author upon request.
